# Novel Green Crosslinked Salecan Hydrogels and Preliminary Investigation of Their Use in 3D Printing

**DOI:** 10.3390/pharmaceutics15020373

**Published:** 2023-01-21

**Authors:** Raluca Ianchis, Rebeca Leu Alexa, Ioana Catalina Gifu, Maria Minodora Marin, Elvira Alexandrescu, Roxana Constantinescu, Andrada Serafim, Cristina Lavinia Nistor, Cristian Petcu

**Affiliations:** 1 National Research & Development Institute for Chemistry and Petrochemistry, ICECHIM, Spl. Independentei No. 202, 6th District, 060021 Bucharest, Romania; 2 Advanced Polymer Materials Group, Politehnica University of Bucharest, 1-7 Polizu Street, 011061 Bucharest, Romania; 3 Collagen Department, Leather and Footwear Research Institute, 93 Ion Minulescu Street, 031215 Bucharest, Romania

**Keywords:** salecan, hydrogels, green crosslinking, biopolymers, 3D printing

## Abstract

Salecan, a kind of polysaccharide, is produced by the Agrobacterium ZX09 salt tolerant strain. In this study, green crosslinked citric acid-salecan hydrogels are explored as novel materials with a high potential for use in regenerative medicine. The impact of salecan and citric acid on the final crosslinked hydrogels was intensively studied and estimated in terms of the whole physicochemical properties and antimicrobial activity. FTIR spectra demonstrated the successful green crosslinking of salecan through its esterification with citric acid where the formation of strong covalent bonds collaboratively helped to stabilize the entire hydrogel systems in a wet state. Hydrogels presented a microporous morphology, good swelling capacity, pH responsiveness, great mechanical stability under stress conditions and good antibacterial activity, all related to the concentration of the biopolymers used in the synthesis step. Additionally, salecan hydrogels were preliminary investigated as printing inks. Thanks to their excellent rheological behavior, we optimized the citrate-salecan hydrogel inks and printing parameters to render 3D constructs with great printing fidelity and integrity. The novel synthesized salecan green crosslinked hydrogels enriches the family of salecan-derived hydrogels. Moreover, this work not only expands the application of salecan hydrogels in various fields, but also provides a new potential option of designing salecan-based 3D printed scaffolds for customized regenerative medicine.

## 1. Introduction

Polysaccharides are the most widespread natural polymers, being found in all living organisms, humans, animals, microorganisms and plants. Owing to their unique biological properties, such as being antioxidant, anti-tumor, antimicrobial, anticoagulant, antidiabetic and antiviral, they are ideal candidates for biomedical applications though they have other uses [1,2]. Polysaccharides, or glycans, are complex biomacromolecules based on monomeric saccharide units bound together by glycosidic bonds. These linkages are formed following the dehydration process of two hydroxyl groups attached to the saccharide units and consist of an oxygen molecule linked between two glycosidic carbon rings [3]. Polysaccharides are actually carbohydrate molecules with long linear or branched chains. When monomeric units of the same kind are connected, long homopolysaccharide chains (homoglycans) result, such as cellulose, starch and glycogen, while different units configurate heteropolysaccharides (heteroglycans), such as hyaluronic acid, heparin and chondroitin sulphate [1].

Salecan is among the lesser known homopolysaccharides. This recently discovered polysaccharide is a special β-glucan originating from the Chinese saline marine environment [4]. Salecan is extracted with the help of the novel salt strain Agrobacterium ZX09, which is a marine microbial polysaccharide. Over the last few years, this polysaccharide has been intensively studied by research groups from China. In the first years after its discovery, salecan was investigated as a dietary supplement, and several studies demonstrated that salecan prevents the digestion process of carbohydrates [5] and fat absorption [6], improving physical performance and resistance to fatigue [7,8], being considered a helpful adjuvant in alcoholic liver disorder [4] and constipation [9]. Moreover, salecan was found to be non-toxic at the concentration used in the test [10], improves gastrointestinal health [11] and has suitable rheological properties to be used as a food additive in the food industry [12,13].

Having a structure rich in hydroxyl groups and promising physicochemical and biological results paved the way for salecan to be used further in association with a variety of monomers/polymers for the synthesis of hydrogels with semi-interpenetrated networks. Unfortunately, solely salecan networks are not mechanically stable, and salecan has been semi-interpenetrated with various synthetic polymers starting from monomers, such as *N*,*N*-dimethylacrylamide (DMAA) and 2-Hydroxyethyl methacrylate [14], where semi-IPN networks with an increased storage modulus resulted with radical polymerization. Moreover, the salecan was imprinted with a thermoresponsive swelling behavior by semi-interpenetrating its networks with poly (N-isopropylacrylamide) [15], and interpenetrated salecan networks with polyvinylalcohol led to an increased cell adhesion compared with the blank synthetic polymer [16], the resulting hydrogels being considered proper materials for application in the biomedical field. Furthermore, the possibility of obtaining oral drug vehicles was studied by grafting the polysaccharide with 2-acrylamido-2-methyl-1-propanesulfonic acid or 3-(methacryloylamino)propyl-trimethylammonium chloride, resulting in pH-sensitive hydrogels able of encapsulating and releasing insulin at pH change [17,18]. Some other semi-interpenetrated networks with salecan were investigated for amoxicillin [19] or doxorubicin release [20,21].

Furthermore, salecan was compounded with polymers from natural resources such as agarose [22], chitosan [23,24], gelatin [25], κ-carrageenan [26], gellan gum [27] or soy protein isolate [28], targeting cell-culture applications, the sustained release of drugs (vancomycin, vitamin C) or envisaging tissue engineering applications, respectively.

However, few hydrogels composed solely of salecan have been synthesized, this fact being closely related to the behavior of the biopolymer because it does not have hydrogel-forming capacity without structural modification. We have identified only three studies in which mostly salecan hydrogels were obtained. Thus, salecan was physically crosslinked through thermal treatment [29] or by using graphene oxide sheets [30], where H bonds were formed, while one study reported ionically crosslinked salecan using chromium ions [31], but in these cases, weak hydrogels were still obtained [29].

The already mentioned studies have their own shortcomings, such as the toxicity of the synthetic polymers used or a reduced stability in wet conditions for a certain period, identified for the resulted samples. Therefore, finding a green method for synthesizing salecan hydrogels with high stability in a wet state is necessary for its use in various fields.

In this context, the main objective of this study was to render a new crosslinked salecan hydrogel using a green crosslinker, namely, citric acid. In recent years, citric acid has been used as a natural crosslinking agent for the preparation of covalently crosslinked hydrogels based on various biopolymers, such as starch [32], pectin, alginate [33], carboxymethyl cellulose [34], chitosan [35] or gelatin [36]. The main advantage of citric acid is its non-toxic nature. Another key feature of citric acid is that it provides valuable antioxidant activity, pH responsiveness and antibacterial properties, being particularly valuable in the synthesis of films and coatings for the food industry (packaging) and cultural heritage protection, but also in biomedical application for tissue engineering applications such as wound dressings [37,38,39].

Citric acid is a bio-polyfunctional aliphatic raw material containing two reactive primary carboxyl groups, an ester-hindered hydroxyl group and a less reactive tertiary carboxyl group. Due to its reactivity with the help of carboxyl groups it can esterify the hydroxyl groups of salecan, which leads to the formation of crosslinking points through the formation of a covalent intermolecular diester bond. The present study aimed to fabricate salecan crosslinked hydrogels and further the prepared hydrogels that are employed as printing inks for the additive manufacturing of 3D constructs. New manufacturing strategies, such as the 3D printing, planned for the current study, are crucial for personalized medicine applications. However, soft materials 3D printing comes with a range of challenges, mostly because of their weaker mechanical strength. In this respect, crosslinked salecan hydrogels are promising materials for additive manufacturing purposes combining the advantages of components and meeting various requirements, including the controllable and tailorable mechanical properties and facile preparation process. Thus, citric acid, as a non-toxic chemical cross-linker, was used to confer mechanical stability to 3D constructs in a wet state and further antibacterial activity. The impact of salecan and citric acid on the final crosslinked hydrogels was intensively studied and estimated in terms of the whole physicochemical properties as well as the antimicrobial activity. Moreover, the rheological properties of salecan-citric acid hydrogel inks were followed as these are relevant for their use in the additive manufacturing process.

The structures of salecan-citric acid hydrogel films were characterized by Fourier transform infrared (FTIR), thermogravimetry analyses (TGA) and a scanning electron microscope (SEM). The swelling ability, pH sensitivity and antimicrobial activity of salecan crosslinked hydrogels were systematically evaluated. The rheological properties of salecan-based hydrogels and the mechanical features of crosslinked hydrogels in a wet state were also evaluated.

This work not only expands on the application of citrate-salecan hydrogels in various fields, but is actually the first time that salecan hydrogels have been investigated for their potential use in additive manufacturing.

## 2. Materials and Methods

### 2.1. Materials

β-1,3-glucan (Salecan) (>90% purity) with an average molecular weight of 2,000,000 g/mol [16,40] was purchased from Suzhou Health Chemicals Co., Ltd. (Souzhou, China) and citric acid (99.5% purity) from SC Remed Prodimpex SRL (Bucharest, Romania). For gel fraction determination, sulphuric acid (95–97% purity) from Supelco (Darmstadt, Germany) and phenol (min. 99.5% purity) from Chimreactiv SRL (Bucharest, Romania) were used. Buffer solutions of pH = 2 (0.1 M HCl (from 37%-sol *w*/*v*, SC Chimreactiv SRL (Bucharest, Romania), pH = 7.4 (phosphate buffered saline (NaCl—0.138 M; KCl—0.0027 M) purchased from Sigma (St. Luis, MO, USA), pH = 11 (0.05 M Na_2_HPO_4_ (Reactivul, Bucharest, Romania) and 0.1 M NaOH (SC Chimreactiv SRL, Bucharest, Romania), were prepared in our laboratory using deionized water.

### 2.2. The Synthesis of Green Crosslinked Salecan Hydrogels

Firstly, we prepared stock solutions of 5%, 10% and 15% *w*/*v* citric acid (CA) in deionized water. Once the stock solutions were obtained, we added the appropriate amount of salecan polysaccharide (S) and mixed it mechanically with a spatula. A blank sample (S0) using deionized water was also prepared. The annotations of the synthesized samples with the specifications of salecan and citric acid concentrations for each sample are presented in Table 1.

The obtained agglomerated gels were kept at 40 °C for 20 h and were occasionally mixed. The resulted hydrated biopolymer hydrogels were centrifuged for 5 min at 9000 rpm, in order to remove the air bubbles. Then, the samples were carefully placed in cylindrical Teflon molds (diameter = 1.6 cm) or cubical silicon molds (1 cm^3^) and were freeze dried. The obtained dried samples were thermally treated for 24 h at 50 °C and further at 130 °C for 20 min. The samples were removed from the oven and stored in desiccators at room temperature.

### 2.3. Physico-Chemical Characterization of Salecan Hydrogels

#### 2.3.1. Determination of the Crosslinking Degree

The phenol-sulfuric acid method was employed to determine the crosslinking degree, where the amount of salecan in the washing solutions of the crosslinked hydrogels was calculated by UV analyses [15,41]. The synthesized rectangular samples (six cubes of each sample) were weighed and placed in 200 mL of deionized water at 45 °C, the uncrosslinked salecan, dissolving very easily in hot water. Every day, a 2 mL sample of washing solution was taken and then the medium was changed with fresh deionized water. It was found that the samples no longer released the unreacted polysaccharide after two days, but for safety, samples from the washing medium were collected for two more additional days. Then, 1 mL of phenol sol 6% *w*/*v* and 5 mL concentrated sulfuric acid were added to the collected washing solution sample and the resulted solution was mixed well. Due to the exothermic reaction, the samples warmed up. Instantly, the change in color was observed, and as the amount of polysaccharide was greater in the washing solutions, the samples prepared for UV tests were more intensely colored in orange. After the samples had been cooled, the wavelength at 490 nm was measured. After that, the absorbance was determined for each sample/for each day and then the mass of cumulated non-crosslinked salecan from the washing solutions was calculated for each sample. Taking into account the mass of salecan in the analyzed sample- m_i_ and the mass of unreacted salecan determined by UV analyses, we have calculated the crosslinked polysaccharide mass- m_f_ and then the crosslinking degree-CD, using Equation (1):

CD salecan % = m_f_ salecan × 100/m_i_ salecan (1)

#### 2.3.2. Swelling Measurements of Salecan Crosslinked Hydrogels

Swelling studies were carried out in different media at 40 °C—deionized water, buffer solutions of pH = 2, 7.4 and 11. The samples were gently removed from the fluids and weighed after a quick dry on filter paper. The samples were weighed from time to time until they reached the equilibrium, and the fluid was replaced periodically with a fresh solution. The swelling degree (ESD) was calculated using the following Equation (2):

ESD (%) = (m_e_ − m_i_) × 100/m_i_ (2)

where m_e_ is the mass of the swollen hydrogel at equilibrium and m_i_ is the mass of the initial dry sample introduced in the medium. The equilibrium swelling degree is reported as the mean value of two determinations.

#### 2.3.3. FTIR Analyses

The FTIR spectra of the salecan crosslinked hydrogels were recorded using Tensor 37 Bruker equipment Fourier transform infrared spectrophotometer equipped with a Golden Gate ATR unit. The samples were analyzed by using ground as-prepared xerogels within the 400–4000 cm^−1^ wave number range.

#### 2.3.4. SEM Analyses

To investigate the morphology of the salecan-based hydrogels, the prepared uncrosslinked hydrogels were quickly frozen using liquid nitrogen and immediately lyophilized. After that, the xerogels were thermocured for salecan crosslinking and were analyzed using SEM. Before testing, all the samples (the thermocured xerogels but also the 3D printed constructs) were sputter-coated with a thin layer of gold to increase their conductivity. SEM images of the samples were acquired using environmental scanning electron microscopy equipment ESEM-FEI Quanta 200 (Eindhoven, The Netherlands).

#### 2.3.5. Thermo-Mechanical Analyses

The thermal properties of the obtained citrate-salecan materials were evaluated using a thermogravimetric analyzer TGA Q5000 (TA instrument, New Castle, DE, USA). Approximately 6 mg of each sample was heated from room temperature to 700 °C and a heating rate of 10 °C/min in a controlled atmosphere with a flow rate of nitrogen of approximately 40 mL/min. The results were processed using TA Universal Analysis.

The mechanical properties of the hydrogel samples swelled in deionized water were achieved using a dynamic mechanical analyzer DMA Q800 (TA Instruments, New Castle, DE, USA). Measurements were conducted at 25 °C to avoid the effects of water evaporation, on round equilibrium swelled samples with a diameter of 15 mm and a thickness of almost 10 mm using a modified compression clamp. In terms of compression analysis, all the samples were compressed with a ramp force of 0.2 N/min, from 0.01 to 18 N. To record the storage (G’) and loss (G”) moduli of the swollen samples, dynamic frequency sweeps were conducted over a frequency range of 0.1–10 Hz with a constant strain of 0.1 % (in the linear viscoelastic region) at 25 °C. The G’ and G” were plotted against the frequency, and tests were repeated three times to ensure reproducibility. The method used to evaluate the hydrogel samples was the compression modulus and frequency sweep, both of which were processed using TA Universal Analysis. All the tests were realized in duplicate.

#### 2.3.6. Determination of Antimicrobial Activity

The antimicrobial assay of the obtained thermocured samples was performed using standard strains from the ICPI, Microbiology Department collection, as follows: *Escherichia coli* (Gram negative) ATCC 11229 and *Staphylococcus aureus* (Gram positive) ATCC 29213. The qualitative screening of the antimicrobial properties was performed using an adapted spot diffusion method [42]. Bacterial suspensions of 1.5–108 CFU_mL^−1^ (corresponding with a 0.5 McFarland standard density) obtained from 24–48 h microbial cultures developed on Mueller Hinton agar (MHA). The plates were left at room temperature to ensure the equal diffusion of the compound in the medium and then incubated at 37 °C for 24 h.

#### 2.3.7. The Rheology Behavior of the Salecan-Citric Acid Hydrogel Printing Inks

The flow behavior of the salecan-citric acid compositions was evaluated through rheological measurements performed with a Kinexus Pro rheometer (Malvern) equipped with a Peltier element for precise temperature control. To obtain information regarding the processability of the formulations, flow curves were registered on a large range of shear rates, from 10^−3^ to 10^3^ at the working temperature (25 °C). A water lock was used to prevent dehydration during the test.

#### 2.3.8. 3D Printing of the Salecan-Citric acid Hydrogel Inks

For the additive manufacturing of the salecan-based hydrogel 3D printed structures, 3D Discovery™ printing equipment (RegenHU Ltd., Villaz-St-Pierre, Switzerland) was used. The additive manufacturing process was performed using a direct dispensing print head. Through BioCAD, soft, rounded and square 3D structures were drawn and the printing process was performed at room temperature. In order to establish the suitable printing parameters, different printing speeds, ranging from 1 to 10 mm/s, and pressures in the 100–300 kPa range, were investigated.

## 3. Results and Discussion

### 3.1. Synthesis and Characterization of Citrate-Based Salecan Biopolymer

#### 3.1.1. The Crosslinking Reaction and the Determination of Crosslinking Degree

**Figure 1:**
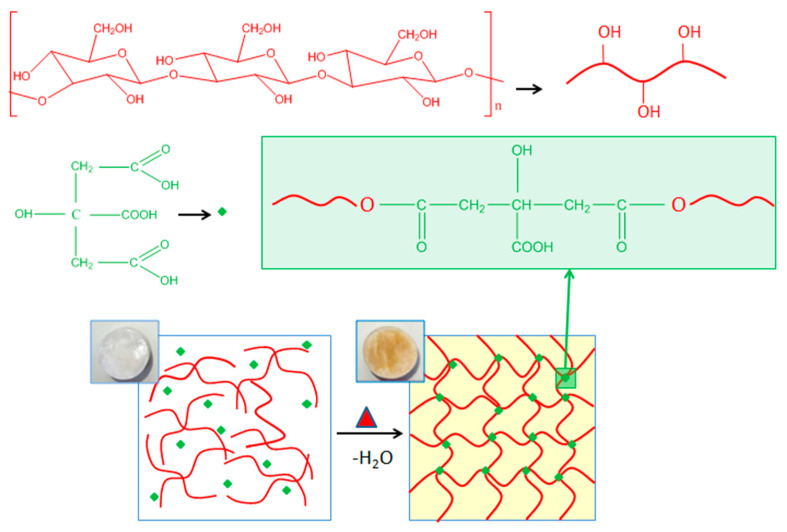
Illustration of the green crosslinking mechanism between salecan biopolymer and citric acid with the resulted citrate-salecan crosslinked networks.

The crosslinking of salecan with citric acid was achieved through the carboxylic functional group of citric acid and the hydroxyl groups of salecan which react via esterification at the elevated temperature of 130 °C [41]. Primary OH groups of polysaccharides may be involved in the esterification of salecan because they are more reactive than secondary OH groups, according to earlier studies [43,44]. A schematic representation of the proposed mechanism is illustrated in Figure 1.

The temperature and time of the crosslinking reaction are directly correlated with the crosslinking degree and, consequently, with the mechanical features and the degradation rate. In this respect, all salecan-based samples were synthesized at the same time, avoiding multiple batches. After synthesis, the thermocured samples were employed for phenol-sulfuric acid method. The amount of polysaccharide dissolved in the washing medium was determined using UV analyses. The results from the UV measurements, which offered information regarding the uncrosslinked salecan, existed in the washing solutions along with the calculated crosslinking degree are presented in Table 2.

The determination of the percentage of salecan chains that were interconnected through citric acid esterification revealed high crosslinking degrees with values ranging from 91 to 96%. These high values suggest that salecan was almost completely crosslinked following the salecan crosslinking reaction and are in good agreement with other studies that mention salecan crosslinking in semi-interpenetrated structures [19,22,28]. It should be mentioned that one sample, S0, containing only salecan, was also prepared and subjected to the crosslinking process at a high temperature. One sample based on salecan-citric acid—the S1 composition was not subjected to thermal shock was also obtained. These samples dissolved when introduced to hot deionized water, thus, confirming the fact that the samples without citric acid, but also the non-thermally treated sample, were not subjected to covalent crosslinking.

Analyzing the degrees of crosslinking of the synthesized hydrogels, differences can be noted depending on the amount of salecan and the crosslinking agent used in the synthesis. Thus, when the hydrogels are obtained in a 15% citric acid solution, the degree of crosslinking increased with the increase in the concentration of salecan, from 91 to 95%, a higher concentration of citric acid favoring crosslinking at high concentrations of salecan. Additions of more biopolymer increased the cross-linked chains at high citric acid concentrations, and consequently, the gelation process improved significantly. Moreover, for the constant polysaccharide concentration of 15%, higher degrees of crosslinking were obtained at increasing concentrations of citric acid, varying from 93 to 95% in agreement with the variation of citric acid from 5 to 15%.

The highest degree of crosslinking was obtained for the low concentration of salecan (5% *w*/*v*) and the lowest concentrations of citric acid (5 and 10% *w*/*v*), 95 and 96%, respectively. Therefore, a low concentration of salecan with a high concentration of citric acid is favorable for the development of less crosslinked hydrogels, probably because too much citric acid blocks the crosslinking of the salecan chains; here, the salecan molecules were rarer anyway. Moreover, for a high concentration of salecan of 15%, we noticed that a concentration of 5% citric acid is not enough, with some biopolymer chains remaining uncrosslinked. For a constant citric acid concentration of 10%, we observed almost insignificant differences in the degree of crosslinking of the hydrogels at various concentrations of salecan, the crosslinked systems being more balanced.

In conclusion, even if the degrees of crosslinking did not vary much between them, there is still a slight dependence of them as a function of the amount of salecan and citric acid used in the synthesis. However, the data demonstrated that by reacting salecan with citric acid, a successful crosslinking was achieved, which leads to permanently covalent bonds as other studies reported for diverse citrate-based polysaccharides [32,36,37]. This fact can be useful when we aim to obtain weaker or stronger hydrogels depending on the envisaged application.

#### 3.1.2. Swelling Properties of Salecan Crosslinked Hydrogels

Generally, the ability of a hydrogel to absorb fluids is influenced by several parameters which compete with each other, such as hydrogel composition, the crosslinking degree, the hydrophilic groups attached to the biopolymer network and the porosity of the hydrogel. Thus, depending on the morphostructure, the polymer retains more or less fluid leading to different swelling degrees. The presence of hydrophilic groups favors the penetration of water into the hydrogel network, thus increasing the degree of swelling. When the penetration of water to the OH groups present in the biopolymer chain is hindered, the degree of swelling is lower.

**Figure 2:**
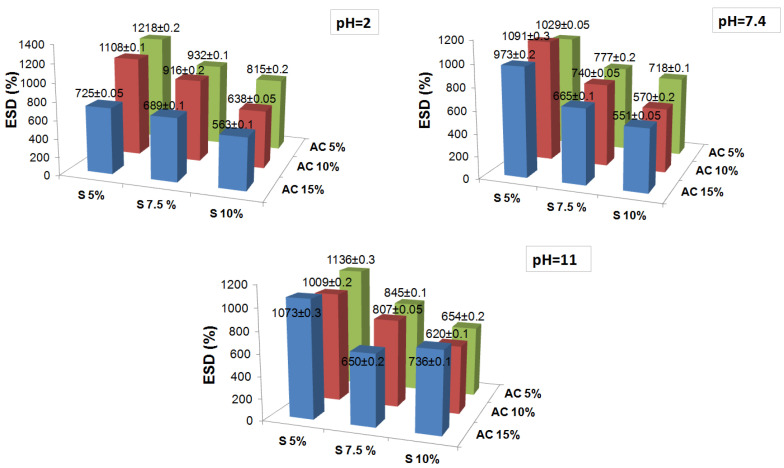
The influence of reactants concentration on the equilibrium swelling degree at different pH values of the obtained crosslinked salecan samples. The results are the average values with added standard errors.

The diverse fluid uptake was followed until the obtaining of a constant weight, which was considered as the maximum or equilibrium swelling degree (ESD). Figure 2 presents the results of the equilibrium the swelling degree obtained in fluids of different pHs for the salecan crosslinked hydrogels. Analyzing the degrees of swelling at equilibrium, relatively high degrees of swelling between 500 and 1200% were obtained. For all fluids, it was observed that the maximum degree of swelling is obtained relatively rapidly (~1 h), probably due to the aerated structure of the hydrogels which caused a rapid penetration of the fluids. After 24 h, the degree of swelling remained quite constant, the samples not retaining significant fluids during this period. It is observed that all samples are stable in fluids for 24 h, after which some of the samples suffer severe modifications. Thus, for the samples kept in deionized water and pH = 2 and 7.4 fluid, the samples presented an insignificant degradation of <10%, even during 30 days, independent of the reactant concentration. The obtained results suggested that strong ester crosslinking achieved using high temperature treatment improved the long-term dimensional and structural stability of the salecan samples in the simulated physiological conditions (pH = 2 and 7.4).

On the other hand, the samples kept in a basic pH medium swell tremendously and disintegrate/dissolve after 24 h, resulting in relatively loose hydrogel structures that are unable to be weighed. As the fluid penetrates, the network loosens and the crosslinked polymer chains break, causing faster fluid penetration resulting in the dynamic change of the swelling degree. The samples obtained at high concentrations of polysaccharide (10%) and high concentrations of citric acid were proved to be the most resistant samples at pH = 11, namely, S2, S3, S4 and S5. Being obtained at a high concentration of polysaccharide with a very well crosslinked network, the biopolymer chains were strongly packed, the fluid penetration was more difficult and the ionizing was slower in the COOH groups until a complete degradation. It is very interesting that the S2, S3 and S4 samples degrade slowly from the outward of the samples, remaining as a slightly yellow tightly packed hydrogel, while S5 swells excessively (~4000%), appearing as a transparent weak hydrogel that very hard to be weighed. Moreover, these hydrogel residues remained as such for almost 7 days until complete degradation, with the S2 and S3 samples being the most resistant to the alkaline media.

At acidic pHs, the samples behaved differently and showed the lowest swelling degrees compared to the other pHs. This is most likely due to the protonation of the OH groups and the formation of hydrogen bonds that cause the networks to tighten and inhibit the penetration of water [45,46]. As the pH increased, the ionization to COO took place, and the electrostatic repulsive forces and the penetration of fluids into the network was favored, leading to an increase in the degree of swelling at pH = 7.4 compared to pH = 2, especially in samples with a lower concentration of salecan. For all fluids, it was noted that the ESD values of all the synthesized samples decreased with the increase in the content of both reactants. This suggested that the hydrogel networks were formed more efficiently at high reactant concentrations. The fluid uptake had been restricted as the network packing increased. Usually, high fluid absorption capacity is desirable in biomedical applications. However, the high fluid absorption capacity could compromise the mechanical strength, which is also necessary for such a field. Thus, an ideal hydrogel for cell culture should possess an adequate swelling capacity, together with excellent mechanical strength.

In summary, the swelling degree can be tailored according to the concentration of the reactants from the initial synthesis step. Moreover, considering that hydrogels have a good swelling capacity as well as pH responsiveness and dependent degradation, they could be of great use in controlled drug delivery in the gastrointestinal tract. The change in pH, could be the trigger for the bioactive agent release by protecting it from the acidic stomach pH and releasing it into the intestines [20,45,46].

#### 3.1.3. Morphological Observations of the Crosslinked Biopolymeric Materials

The materials porosity is extremely important when envisaging biomedical applications, as it is an essential aspect for controlled release of bioactive agents, wound dressing efficacy as well as for proper regeneration as they work as a support and guide tissue formation [47].

**Figure 3:**
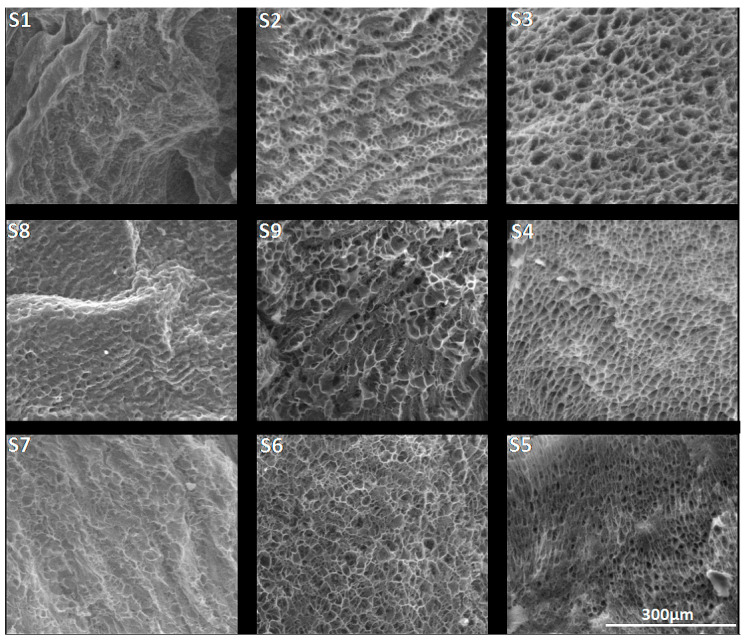
SEM images of the synthesized crosslinked citrate-salecan xerogels (magnitude 500×).

SEM images, presented in Figure 3, revealed a porous morphology for all the synthesized salecan samples. The samples presented interconnected macropores with mostly rounded or elliptic shape. Generally, the distribution of the pores is quite uniform, for the samples with high concentration of polysaccharide, the thin walls that separate the voids being more evidenced and apparently with higher dimensions as those obtained at low reactant concentrations. As the concentrations of salecan and citric acid have increased, the contact between the polymer chains was more likely, the polysaccharide chains grew following crosslinking and thus the formation of a stable uniform structure was favored. However, as the concentration of citric acid increased, the samples obtained at low polysaccharide concentrations revealed smaller and less defined pores. This may also be due to the presence of a large amount of citric acid that can clog the pores at lower polysaccharide concentrations.

As we mentioned before, the porosity of the samples can affect the swelling capacity of the hydrogels. Taking into consideration the reduction of the swelling degrees at high reactant concentrations, we assume that the increase of the specific surface area of the hydrogel samples is the determinant factor for the samples, which presented smaller pore sizes and higher swelling degrees [48]. Large specific surfaces of small pores could allow the retention of more fluid due to the higher contact surface between the material and the fluid [49,50]. Certainly, the fluid uptake can be governed by the chemical structure of the samples, their morphology, meaning the porosity together with the specific surface of the pores, or more likely by an input of each.

#### 3.1.4. FTIR Analyses

In order to evidence the influence of the concentrations of citric acid, as well as salecan’s influence on the structural properties of the final synthesized crosslinked materials, we have considered the selection of the most relevant samples.

**Figure 4:**
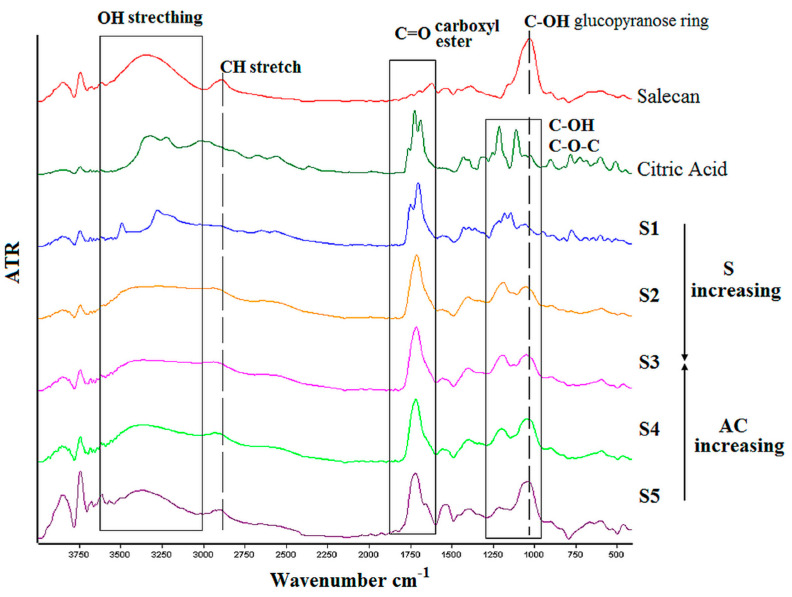
FTIR spectra of the crosslinked citrate-salecan samples.

FTIR curves corresponding to the samples obtained at the highest concentration of crosslinker with the three concentrations of polysaccharide (S1, S2, S3) and in the same manner, the highest concentration of salecan in the presence of the three concentrations of citric acid (S3, S4, S5) are superimposed in Figure 4, along with salecan and citric acid spectra. FTIR spectra of salecan presented the characteristic absorption band of COH, stretching at 1018 cm^–1^, which corresponds to the glucopyranose ring from the polysaccharide structure. Peaks at around 2900 cm^−1^ were attributed to the C-H stretching vibrations while the peaks from 3000–3600 cm^−1^ range belong to the OH stretching frequency [28,41,46]. Citric acid spectra displayed a split peak at ~1700 cm^−1^, which corresponds to COOH from the C=O stretching vibration, peaks from 1200 cm^−1^ attributed to C-O-C bonds, along with peaks from 2800–3300 cm^−1^ region, which are associated with OH and CH stretching vibration, respectively [36,37].

All these characteristic peaks for salecan and citric acid existed in the spectrum of salecan crosslinked hydrogels but, as expected, with some modifications and shifts in the wavenumbers values. For instance, with increasing salecan and citric acid concentration, the peak corresponding to the OH stretching broadens in the case of crosslinked hydrogels. Such broadening is the results of salecan OH groups’ condensation with the COOH functional groups of citric acid with the formation of acetal bridges after crosslinking.

Additionally, modifications in the 1700 cm^−1^ region revealed the formation of typical ester carbonyl groups from the esterification reaction. The most important, the intensity of the absorption band of carbonyl group varies as function of salecan and citric acid concentration namely, the peaks are shifted and sharper with the increase of citric acid concentration at constant salecan amount and the same, sharper at constant citric acid with increased concentration of salecan. This was attributed to a more crosslinked structure resulted when the samples are exposed to high temperature and with subsequent crosslinking reaction. The shifting and modifications of peaks observed at approximately 1000–1200 cm^−1^ in the crosslinked hydrogels may be attributed to C-OH and C-O stretching vibrations during the formation of crosslinks [37,51].

Briefly, the modifications evidenced by the FTIR spectra demonstrated the successfully green crosslinking of salecan through its esterification with citric acid where the formation of strong covalent bonds collaboratively helped to stabilize the entire hydrogel systems.

#### 3.1.5. Thermomechanical Analyses of Salecan Crosslinked Materials

**Figure 5:**
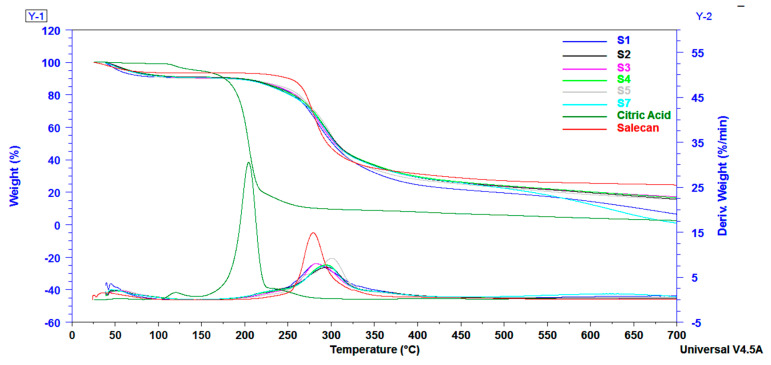
TGA-DTG curves of salecan, citric acid and the crosslinked salecan materials.

TGA analysis provided a convenient way to study the thermal properties of salecan and the synthesized citrate-salecan materials. The thermal behavior of the samples was reflected by the TG and DTG curves which are presented in Figure 5. As shown in Figure 5, all salecan samples presented three distinct decomposition stages. The first stage, ranging from 25 °C to 150 °C, resulted in a weight loss of ~9%, occurred for all citrate-salecan-based samples and was associated to the evaporation of the absorbed water and slightly volatile substances [30]. The most significant weight losses were registered for the second (~65–68%) and third decomposition (8–25%) steps found between 150–450 °C and 450–700 °C, respectively. These stages were related to a complicated breakage and decomposition process involving the destruction of the strong covalent crosslinks but also the fragmentation of the polysaccharide backbone, including the rupture of the C–O and C–C bonds of the saccharide ring [31,52].

The crosslinked samples showed an extra shoulder peak at ~220 °C due to an excess of citric acid. The endothermic maximum decomposition peak displayed at 280 °C for salecan presented modifications in the DTG curves registered for the citrate-salecan samples, depending on the amount of citric acid used in the synthesis. Thus, the samples with a higher citric acid concentration did not display improved thermal stability, while samples with a low concentration of crosslinking agent, such as samples S5 and S7, showed an increased decomposition temperature with an additional 15–20 °C. This fact denotes that citric acid improved the thermal stability of the samples, but an excess of citric acid does not imply an increased thermal stability, as proved in earlier reports [37].

**Figure 6:**
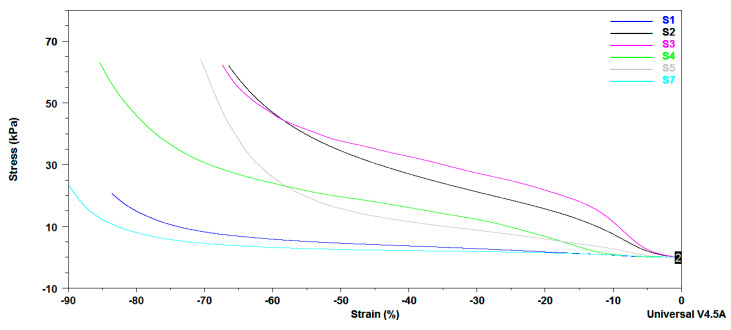
DMA stress–strain curves obtained for the crosslinked salecan samples swollen in deionized water at equilibrium.

DMA mechanical properties of citrate-salecan-based hydrogel samples are presented in Figure 6. Earlier studies demonstrated that the presence of salecan induced changes in the mechanical properties of several hydrogels. As expected, the mechanical properties were enhanced with increasing salecan but also citric acid concentrations in the biopolymeric matrix. The addition of more salecan and citric acid enables more chain interaction resulting in a denser crosslinked network through covalent bonding, leading to mechanical improvements. Thus, DMA curves suggested that samples S3 and S2 presented a better resistance to stress conditions, followed by S4 and S5. Instead, S1 and S7 samples, which have a low content of both salecan and citric acid, have a weaker response to mechanical stress. These results correlate very well with swelling analyses where the samples rich in biopolymers (S2–S5) presented a lower fluid uptake than the samples obtained at low reactant concentrations (S1, S7), the fluid contents influencing the mechanical properties of the samples in a wet state.

It is worth mentioning that all the samples recovered immediately after the applied mechanical stress, presenting greater elasticity (see supplementary video). After analyses, the water expelled under stress conditions was rapidly reabsorbed, the samples rapidly regaining their initial forms.

**Figure 7:**
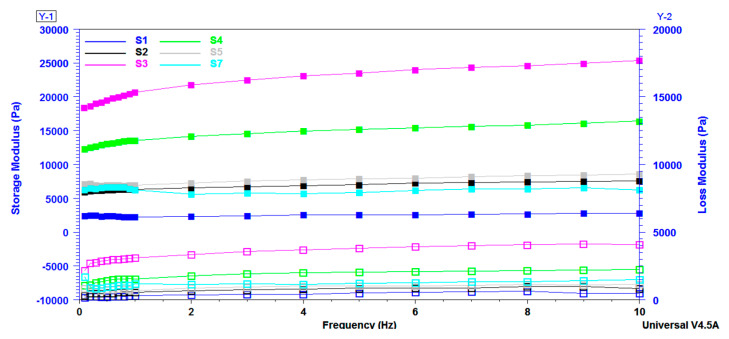
Storage modulus (G’) and loss modulus (G’’) as a function of frequency registered for salecan crosslinked hydrogels.

Frequency sweep method was used to measure the storage modulus G’ and loss modulus G” of the equilibrium swelled samples. These two moduli are registered as a function of the frequency of crosslinked citrate-salecan-based hydrogels and were represented in Figure 7. It is important to mention that G’ is greater than G” over the entire 0.1–10 Hz frequency domain. As a result, loss modulus covered a range of 10 Pa to 4000 Pa, while storage modulus varied between 2500 and 25,000 Pa. This fact indicates the crosslinked behavior of the salecan hydrogels prepared with citric acid [53,54]. Moreover, these results indicated that the elastic character of the synthesized hydrogels ruled over the viscous character [55].

The values of the two viscoelastic moduli increased with the concentration of reactants, showing that more rigid hydrogels were developed due to the higher content of biopolymers, which determined a higher proportion of entangled polymer chains and a lower degree of swelling. The hydrogels displayed a 6-fold increase in G’ and a 10-fold increase in G” at the frequency of 1 Hz when the salecan concentration was doubled (S1 to S3), while they displayed a ~2.5-fold increase in G’ and a ~3.5-fold increase in G” when the citric acid concentration was tripled (S5 to S3).

It should be noted that the samples presented a great stability when the frequency was increased, which is very important when the material is designed for applications subjected to mechanical demands.

#### 3.1.6. Antimicrobial Activity of Green Crosslinked Salecan Hydrogels

**Figure 8:**
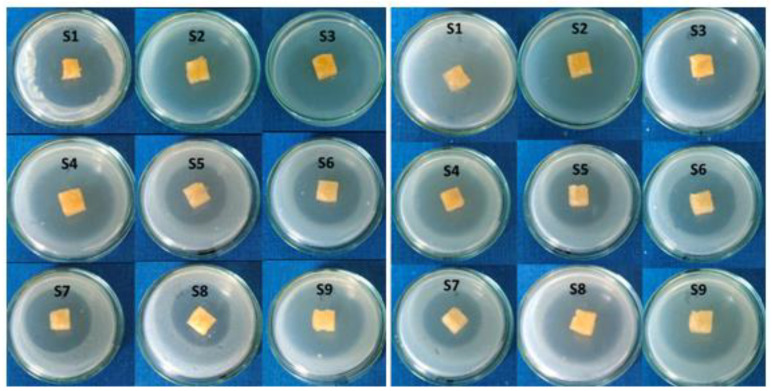
Antimicrobial activity against *Escherichia coli* (**left side**) and *Staphylococcus aureus* (**right side**).

All tested citrate-salecan hydrogel samples presented a good antibacterial activity against the two tested strains, *Escherichia coli* and *Staphylococcus aureus*. Sensitivity was evaluated by measuring the diameters of the inhibition zones which appeared around the spot and were expressed as follows: “−” absence of clear inhibition zone; “+/−” very weak zone of inhibition; “+/−” weak zone of inhibition and “+” clear zone of inhibition. As measured, the zone of inhibition varied from 17 to 35.5 mm (Figure 8). The differences in the inhibition zone for all the samples were influenced by citric acid concentration, as can be observed in Table 3.

The difference between the two values of the degree of inhibition for the two types of bacteria tested can be given by the structure of the cell wall. *Escherichia coli* is a Gram-negative bacterium, unlike *Staphylococcus aureus*, which is a Gram-positive bacterium with a more resistant cell wall due to the basic unit.

The highest inhibition area was shown by samples S1 and S2, which have the highest content of citric acid for the both tested strains. The results are in good agreement with other studies that demonstrated the ability of citric acid to act as a antimicrobial agent [37,38,39].

### 3.2. Preliminary Investigation of Salecan Hydrogels for 3D Printing Purposes

#### 3.2.1. The Rheology of Salecan-Based Hydrogels Used Further as Printing Inks

**Figure 9:**
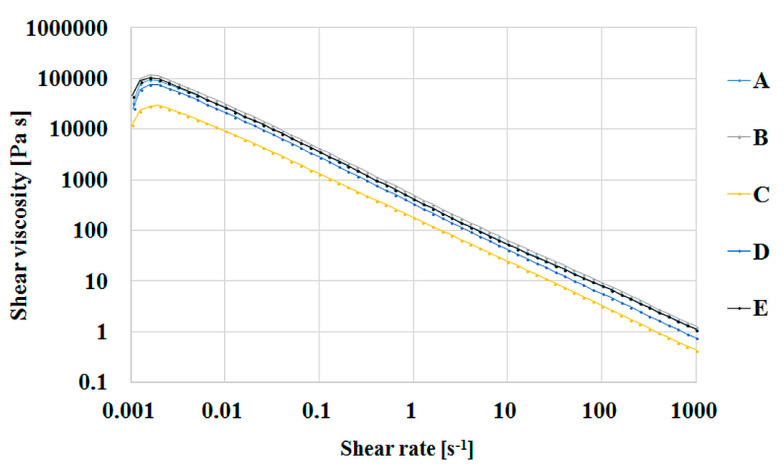
Shear viscosity as function of shear stress for salecan-citric acid hydrogels where A-S5; B-S4; C-S1; D-S2; E-S3.

As expected, the viscosity of the salecan-based hydrogels increased with the increasing polysaccharide concentration at a constant citric acid concentration. As shown in Figure 9, the rheological behavior of compositions S1–3 is identical, but the viscosity of S3 increases approximately 10 times with an increase in salecan concentration from 5% to 10%. Increasing the concentration of citric acid in the compositions with 10% salecan preserves the pseudoplast character of the polysaccharide and very slight changes in the viscosity value were registered. Taking into account that the hydrogel samples were analyzed after the hydration step (20 h at 40 °C), it is possible to assume a pre-crosslinking stage occurred during this period. However, the influence of the citric acid amount on viscosity is rather minor and predictable for a small molecule with high solubility in water. The samples with a low concentration of salecan and with a low concentration of citric acid show the lowest values of viscosities, while the samples with a high concentration of polysaccharides and citric acid show the highest values of viscosities.

Thus, all compositions showed a shear thinning behavior in the studied shear rates interval. Viscosity in the related series increased with increasing citric acid and salecan concentrations. This rheological behavior is common for polysaccharide solutions and is attributed to their large hydrodynamic size, resulting from the association of linear and stiff macromolecules, leading to high viscosities and pseudoplasticity [56]. Moreover, the viscosity of salecan hydrogels decreased from ~10^5^ Pa·s to 10 Pa·s, with an increasing shear rate, exhibiting shear-thinning behavior and indicating the possibility of injecting salecan hydrogel-based inks [57].

Furthermore, the results are in good agreement with other salecan studies, which evidenced the same non-Newtonian viscosity behavior for salecan hydrogels, its excellent rheological properties recommending it as a new source of thickening agent [13,27,29].

#### 3.2.2. Preliminary Investigation on the Printability of Salecan Hydrogels

**Figure 10:**
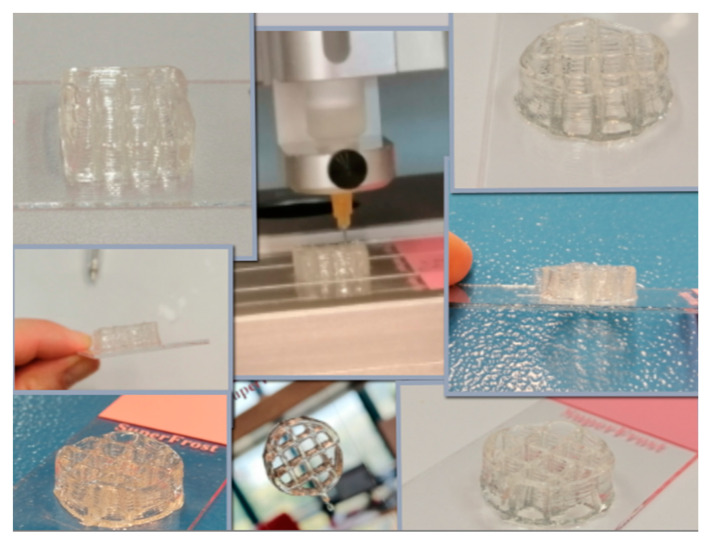
3D Printing constructs manufactured using salecan hydrogel inks (selected images for 10% wt salecan formulations).

**Figure 11:**
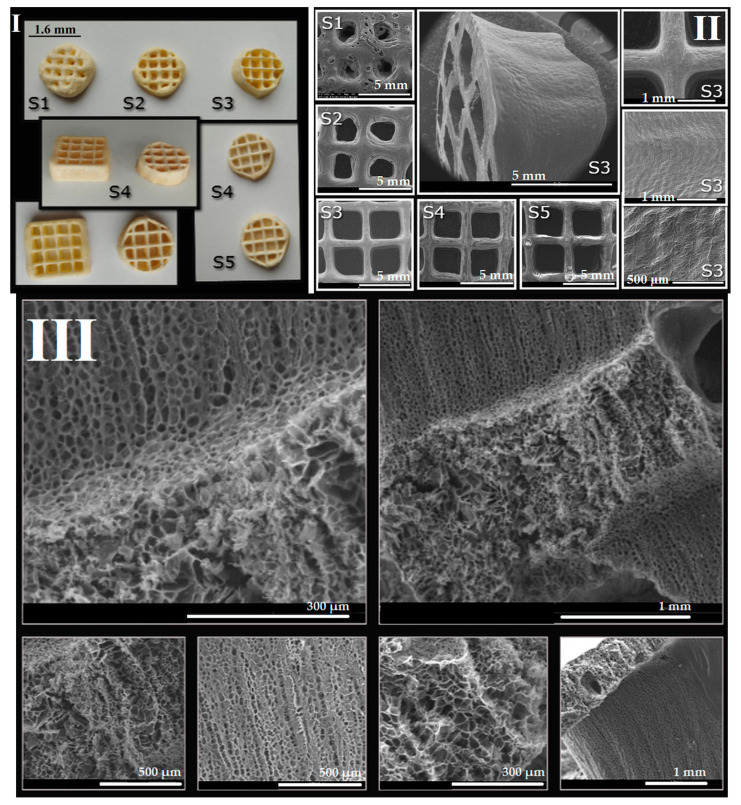
(**I**). The appearance of the lyophilized 3D printed salecan-based constructs after thermocuring. (**II**). SEM images of the 3D printed samples with an emphasis on the macropores; (**III**). SEM images collected on the surfaces of the fractured 3D printed sample (S4).

Different concentrations of salecan were investigated to verify the possibility of their use in the additive manufacturing process, starting with low concentrations of biopolymer up to very high concentrations. In order to preserve the printed shape, the 3D printed structures were frozen and then dried by lyophilization. It was found that at low concentrations of salecan, up to 3–5 printed layers can be reached, but even so, the obtained structures collapse during printing, the initial structural integrity being seriously affected. This fact is due to the large amount of water in the hydrogel, but also to the fact that biopolymer networks cannot support such a weight. As the concentration of salecan was increased, the viscosity of the paste was greater; consequently, the number of printed layers increased as well as the fidelity and integrity of the 3D printed structures. The mechanical stability was greatly improved at concentrations of 7.5–10% *w*/*v* salecan, and the 3D printed forms were highly stable during printing and reached up to 15–20 layers without collapse. Instead, due to the high viscosity, higher pressures are used for needles with smaller diameters (up to 300 KPa) or needles with a thicker diameter (0.25–0.33 mm) to maintain low pressures causing changes in the finesse of the 3D constructs. Some 3D printed constructs manufactured using 10% wt salecan hydrogel inks are presented in Figure 10. Regardless of the citric acid content, all of the hydrogels containing 10% wt salecan were very successfully printed, producing 3D constructs with very identical shapes and macroporous structures as also presented in Figure 11.

The printing speed is also an important parameter and we found its dependence on the concentration of the printing paste. Thus, we can have high printing speeds of ~10–12 mm/s at lower concentrations of biopolymer, the printing paste rendering continuous filaments. As the concentration of the paste increases, the printing speed must be reduced (1–4 mm/s) due to the fact that the contact time between the printed filament and the already printed one must be increased to ensure the adhesion of the hydrogel layers to each other.

One thing worth taking into account was the fact that, even if the speed is limited to 1 mm/s, at concentrations higher than 10%, the layers do not easily stick to each other and even hang the layers already deposited. Also, it is very hard to obtain continuous filaments when salecan hydrogel is extruded through the nozzle. Thus, pastes with too high concentrations of salecan (over 10%) render 3D constructs with some defects.

Also, the printing time is very important when dealing with printing inks rich in water, such as the hydrogels. As the printing process took place, the water in the already deposited filaments began to evaporate, the base of the printed shapes narrowed, and irregular shaped 3D printing constructions resulted. (see Figure 11, SEM images on rounded shape). Therefore, the printing process must be optimized starting from the size of the needle, the printing speed, the printed form and the number of layers so that we can obtain regular forms with high fidelity and integrity. Based on the collected results, we can single out the use of 10% salecan hydrogels with various citric acid concentrations as the most promising salecan hydrogel printing inks.

#### 3.2.3. The Appearance of the 3D Printed Salecan-Based Structures and Their Morphology

Tissue substitutes should have an appropriate morphostructure and reproduce as much as possible the local microenvironment of the intended site. A suitable porosity of the biomaterial support, but also a proper nutrient feeding, could ensure the proliferation of cells in good conditions [47].

Thus, we have successfully obtained 3D printed constructs using salecan hydrogels which were freeze dried and thermocured. Some of the thermocured printed constructs are presented in Figure 11. The printed constructed have acquired a yellowish color after the thermal treatment. The printed constructs obtained from hydrogels with high concentrations of salecan presented better defined structures, compared to the constructs obtained at lower concentrations of the biopolymer (S1 and S2). For the S3–S5 samples, the shape of the structure preserved better their macropores during and after printing, the corresponding hydrogel inks being richer in biopolymer content.

SEM images offered some helpful information regarding the morphological features of the 3D printed salecan-based scaffolds. Thus, we can observe the fact that at low concentrations of salecan, the printed samples show mostly rounded macropores. At higher salecan amounts the macropores changed to more rectangular shapes corresponding very well with the predefined shape established through the 3D printer soft. The deposited layers were greatly welded but still well-defined; thus, a layered morphology of the 3D printed shape at high concentration of salecan was revealed. As mentioned previously, due to the low printing speed used for viscous solutions with high concentrations of biopolymer, the base of the printed form starts to dry during the printing of the upper layers. Consequently, the final shape of the 3D structure becomes slightly conical as evidenced from the SEM images, too (Figure 11, S3).

The internal morphologies are extremely spectacular, as disclosed from the SEM images collected on the fractures of the 3D printed samples (for the S3 and S4 samples). The images highlighted the presence of interconnected micropores of different sizes and shapes. Essentially, the porous morphology provides large specific surfaces, further needed for the encapsulation and controlled release of various bioactive agents highly beneficial in the field of tissue engineering.

## 4. Conclusions

In the present research, we have demonstrated that the formation of biopolymer crosslinked networks played a key role in the stability of the hydrogel in a wet state. Moreover, by controlling the reactant concentrations, we have facilitated the selective absorption of fluids in various pH media thanks to the preserved microporous morphology. Furthermore, the strong covalently cross-linked salecan hydrogels synthesized by the present solvent-free green process possessed versatile characteristics (e.g., stability in physiological environment, responsiveness to pH stimuli, great mechanical stability) which have tremendous potential for diverse biomedical applications. Our preliminary findings also have a great impact on the possible applications of salecan in the 3D printing area for regenerative medicine purposes.

More investigations are needed to better understand the interactions between the biopolymers used and how their concentration could influence the printing process but also the physical-chemical and biological properties of the 3D printed structures. Future perspectives include testing crosslinked hydrogels for biological response but also the trapping of bioactive substances in the structure of 3D printed constructs.

## Figures and Tables

**Table 1:** Samples annotations and feed compositions of the synthesized salecan hydrogels.

Sample Name	Composition	Salecan (g)	Medium (mL)	CA Concentration (%)
*S0*	*S10%*	*1*	*10*	-
**S1**	S5-AC15	0.5	10	15
S2	S7.5-AC15	0.75	10	15
**S3**	S10-AC15	1	10	15
S4	S10-AC10	1	10	10
**S5**	S10-AC5	1	10	5
S6	S7.5-AC5	0.75	10	5
**S7**	S5-AC5	0.5	10	5
S8	S5-AC10	0.5	10	10
S9	S7.5-AC10	0.75	10	10

**Table 2:** The concentration of salecan in the washing solution determined by UV-Vis measurements and the calculated crosslinking degree.

Sample Name	Concentration × 10^−3^ (mg/mL)	CD (%)	STD
S1	0.023	91	±0.03
S2	0.028	93	±0.02
S3	0.021	95	±0.04
S4	0.025	94	±0.03
S5	0.034	93	±0.02
S6	0.016	95	±0.04
S7	0.009	96	±0.03
S8	0.012	95	±0.04
S9	0.017	95	±0.02

**Table 3:** The evaluation of the antimicrobial activity of the citrate-salecan samples.

Sample	*E. coli* ATCC 11229 (Gram Negative)			*S. aureus* ATCC 29213 (Gram Positive)		
	Evaluation	Inhibition Zone (mm)	SD (Standard Deviation) for Three Determinations	Evaluation	Inhibition Zone (mm)	SD (Standard Deviation) for Three Determinations
S1	+	30	0.1	+	35	0.2
S2	+	35.5	0.1	+	33	0.2
S3	+	27.5	0.2	+	29.5	0.1
S4	+	26	0.1	+	27.5	0.1
S5	+	22.5	0.1	+	25	0.2
S6	+	19	0.2	+	21	0.1
S7	+	22	0.1	+	18	0.1
S8	+	17	0.1	+	21	0.1
S9	+	29	0.2	+	27.5	0.1

## Data Availability

Not applicable.

## Supplementary Materials

The following supporting information can be downloaded at: https://www.mdpi.com/article/10.3390/pharmaceutics15020373/s1, Video S1: DMA analyses on swelled salecan hydrogels.
